# Intra-individual structural covariance network in patients with chronic neck and shoulder pain: a longitudinal brain structure analysis

**DOI:** 10.3389/fneur.2026.1732732

**Published:** 2026-02-20

**Authors:** Tianci Liu, Zhiqiang Qiu, Jia Ming, Maojiang Yang, Libing He, Hongjian Li, Xiaoxue Xu

**Affiliations:** 1 Department of Radiology, Affiliated Hospital of North Sichuan Medical College, Nanchong, China; 2 Department of Pediatrics, Affiliated Hospital of North Sichuan Medical College, Nanchong, China; 3 Department of Pain, Affiliated Hospital of North Sichuan Medical College, Nanchong, China

**Keywords:** chronic neck and shoulder pain, graph theory, intra-individual structural covariance network, longitudinal analysis, minimally invasive intervention

## Abstract

**Background:**

Neuroimaging studies have suggested that the neural mechanisms underlying chronic neck and shoulder pain (CNSP) are associated with morphological alterations in various cortical regions. However, there is a scarcity of research exploring the structural network characteristics of the brain in patients with CNSP. While most existing studies focus on group-level brain structural networks, there is a lack of insight into individual variability. Additionally, longitudinal studies investigating changes in the brain's structural networks following treatment in CNSP patients remain limited.

**Methods:**

To address these gaps, this study enrolled 25 patients with CNSP, obtaining structural brain MRI data and clinical measures before treatment and 3 months after a minimally invasive intervention. Individual-level structural covariance networks were constructed for each participant to explore structural network differences between CNSP patients and healthy controls (HCs). Longitudinal changes in these networks were also assessed post-intervention.

**Results:**

Compared to HCs, CNSP patients exhibited significantly reduced Degree Centrality (*P* = 0.03, FDR corrected) and Nodal Efficiency (*P* = 0.0082, FDR corrected) in the right inferior frontal gyrus (pars triangularis), with significant structural recovery observed 3 months after the intervention. In terms of global network topology, the CNSP group showed decreased small-world properties, specifically in Gamma (*P* = 0.0093) and Sigma (*P* = 0.0301) indices; however, unlike local metrics, no significant recovery was observed in these global metrics 3 months post-intervention. Furthermore, correlation analysis demonstrated a significant negative association between the baseline Degree Centrality of the right inferior frontal gyrus and the percentage change in VAS scores (*r* = −0.47, *P* = 0.0168, FDR corrected), suggesting a potential prognostic value.

**Conclusion:**

These findings provide valuable longitudinal data that help elucidate the central mechanisms of pain in CNSP patients. They also identify potential biomarkers that could predict the response to minimally invasive interventions, offering insights into individualized treatment strategies for chronic cervical and shoulder pain.

## 1 Introduction

Chronic neck and shoulder pain (CNSP) is a predominant clinical indicator of cervical spondylosis, typically defined as ongoing pain in the neck and shoulder areas persisting for more than 3 months (1). Globally, CNSP demonstrates a high prevalence. A comprehensive systematic review reported that the annual prevalence ranges from 16.7 to 75.1%, with a pooled estimate of 37.2%, while lifetime prevalence was estimated at 48.5% (2). In the United States, neck pain ranks as the fourth most significant contributor to disability, following closely behind lower back pain, depression, and other musculoskeletal issues (3). Furthermore, recent health economic analyses reveal that, out of 154 assessed conditions in the U.S., neck and lower back pain are among the top drivers of healthcare expenditure, with estimated costs reaching $134.5 billion annually (4).

Resting-state functional magnetic resonance imaging (rs-fMRI) has been extensively utilized to map the functional landscape of Chronic Neck and Shoulder Pain (CNSP) (5). Previous studies have successfully identified functional connectivity disruptions in key pain-processing hubs; for instance, enhanced connectivity between the amygdala and prefrontal cortex has been linked to central sensitization (6), while alterations in periaqueductal gray (PAG) networks correlate with pain modulation deficits (7). However, while rs-fMRI effectively captures these dynamic fluctuations in brain activity, it primarily reflects the physiological state of pain processing. Chronic pain is not merely a functional disorder but a condition that drives long-term neuroplasticity, eventually manifesting as structural maladaptation—specifically, alterations in gray matter (GM) morphology. To date, how these functional network disruptions translate into stable structural network reorganization remains largely unexplored. Therefore, investigating structural covariance networks based on GM morphology is essential to complement rs-fMRI findings, offering a window into the cumulative, long-term neuroanatomical consequences of CNSP.

Therefore, researchers have begun to investigate the changes in brain gray matter structure in patients with CNSP. Research has demonstrated that patients with CNSP exhibit various morphological changes in the cerebral cortex, including alterations in cortical volume, thickness, and sulcal depth (8, 9). Specifically, De Pauw et al. reported distinct patterns of structural neuroplasticity depending on the etiology of neck pain: compared to HCs, patients with chronic whiplash-associated disorders (CWAD) showed a reduction in cortical volume in the right precentral gyrus and superior temporal gyrus, whereas patients with chronic idiopathic neck pain (CINP) exhibited increased cortical volume in the left superior parietal lobule and cortical thickening in the left precuneus and left superior parietal lobule. Furthermore, these structural alterations were significantly correlated with deficits in motor performance (e.g., neuromuscular control and strength) (8). In a study by Niddam et al. (9), who utilized surface-based morphometry techniques, it was found that patients with chronic shoulder pain exhibited shallower sulcal depths in various brain regions, including the right central sulcus, posterior insula, inferior frontal gyrus, dorsomedial prefrontal cortex, precuneus, temporal cortex, and the left medial orbital frontal cortex. Notably, the depth of the right central sulcus was negatively correlated with pain intensity, while the depth of the left medial orbital frontal cortex showed a negative correlation with pain-related emotional responses.

Some studies have employed graph theory analysis to investigate changes in the structural covariance network topology in patients with chronic low back pain. Researchers computed the pairwise correlation coefficients of morphological measures within regions of interest (ROIs) to generate a group-level structural covariance network for each cohort. The results revealed that, compared to HCs, patients with chronic low back pain exhibited lower global efficiency, smaller small-worldness, and longer characteristic path lengths in their structural covariance networks, indicating impairments in brain network integration and processing (10). However, although this study described the brain network at the group level in chronic pain patients, it did not capture individual-level variability. To address this limitation, Yun et al. (11) proposed an individual structural covariance network, which allows for the creation of a personalized brain network for each individual within the group. While prior longitudinal research has demonstrated partial reversal of brain structural and functional abnormalities following treatment in chronic pain populations (12–14) it remains unclear whether the specific topological abnormalities in the structural covariance network of CNSP patients can be restored following minimally invasive intervention. Furthermore, the potential predictive value of baseline structural network features for treatment outcomes in this specific population has yet to be fully elucidated.

To address the aforementioned gaps, this study aimed to construct individual-level structural covariance networks to investigate the structural network differences between CNSP patients and HCs, as well as the longitudinal changes following intervention. Additionally, the study analyzed the relationship between baseline structural network abnormalities in CNSP patients and treatment outcomes. The goal is to provide a reference for predicting the efficacy of minimally invasive treatments based on individual baseline structural network abnormalities in patients with CNSP, and to offer neuroimaging evidence for personalized and precise treatment strategies.

## 2 Methods

All research procedures were approved by the Ethics Committee of the Affiliated Hospital of North Sichuan Medical College and were carried out in strict adherence to the ethical principles outlined in the Declaration of Helsinki (Approval No. 2023ER95-1). Prior to participation, all participants provided written informed consent after being fully briefed on the study's purpose, procedures, and potential risks.

### 2.1 Participants

CNSP Group: Participants in the CNSP group were diagnosed with CNSP by two experienced pain specialists at the Affiliated Hospital of North Sichuan Medical College, according to the chronic pain diagnostic criteria established in the 11th revision of the International Classification of Diseases (ICD-11) (15). The inclusion criteria were as follows: (1) Persistent neck and shoulder pain, with or without radiating pain to one or both upper limbs, lasting for a minimum of 3 months, accompanied by radiographic evidence of cervical spine degeneration on X-ray or MRI; (2) Patients exhibiting resistance to pharmacological treatments or experiencing significant adverse drug reactions, and who were scheduled to undergo minimally invasive interventional therapy for cervical intervertebral disc pathology [collagenase-induced chemonucleolysis combined with ozone injection (16)] at the Pain Medicine Department of our hospital; (3) Absence of clinically significant pain in other regions of the body; (4) Age between 20 and 70 years, right-handed; (5) No contraindications for MRI scanning. Exclusion criteria: (1) Presence of major intracranial pathologies, such as extensive cerebral infarction, encephalomalacia, or brain tumors; (2) Diagnosis of primary psychiatric disorders, including but not limited to anxiety, depression, Alzheimer's disease, schizophrenia, or other neurological or psychiatric conditions; (3) Presence of severe systemic comorbidities, such as advanced cardiac, hepatic, or renal dysfunction.

HCs Group: The inclusion criteria for the HCs group were: (1) Matched to the CNSP group in terms of age and handedness; (2) No contraindications to MRI scanning; (3) Absence of any acute or chronic pain symptoms. Exclusion criteria: (1) Presence of significant intracranial abnormalities; (2) A history of neurological or psychiatric disorders.

### 2.2 Clinical indicators assessment

Clinical indicators for all participants were assessed within 1 h prior to each of the two MRI scans. Pain intensity over the preceding week was quantified using the Visual Analog Scale (VAS) (17), with scores ranging from 0 (representing no pain) to 10 (indicating the most intense pain imaginable). The duration of pain was defined as the period between the initial diagnosis of CNSP and the date of the preoperative brain MRI.

### 2.3 Imaging acquisition

All MRI imaging was performed using a Siemens MAGNETOM Skyra 3.0T MRI scanner, equipped with a 20-channel head-neck combined coil. Patients diagnosed with CNSP underwent two scans: one within 24 h before treatment and the second 3 months after the minimally invasive procedure. During the scans, participants were placed in a supine position on the MRI table, and foam padding was used to stabilize their heads and reduce movement. To minimize external noise, earplugs were provided. Patients were instructed to remain as still as possible throughout the scan.

For structural imaging, high-resolution T1-weighted images were acquired using a 3D Magnetization Prepared Rapid Gradient Echo (MP-RAGE) sequence. The scan parameters were as follows: repetition time = 2,240 ms, inversion time = 1,130 ms, field of view = 256 × 256 mm, data matrix = 256 × 256, slice thickness = 1 mm (without gap), number of slices = 192, and voxel size = 1.0 × 1.0 × 1.0 mm.

### 2.4 Data preprocessing

The raw DICOM fileswere first converted to NIfTI format using MRIcroGL. The Recon-all process includes:The images were normalized and registered using the Talairach and Tournoux atlas, followed by correction for magnetic field inhomogeneities and skull stripping, resulting in segmentation into gray matter, white matter, and cerebrospinal fluid. Subsequently, the two surfaces of the white matter and the pial layer were reconstructed (18), and the cortical thickness map was obtained by calculating the distance between the two surfaces, applying a Gaussian kernel with a 10 mm width for smoothing.

Following this, the Desikan-Killiany atlas (19) was used to segment the cortical regions of the brain, resulting in 68 regions across the left and right hemispheres. FreeSurfer software was then employed to automatically calculate the cortical thickness for each region (18). In addition, seven subcortical regions were included, namely the thalamus, caudate nucleus, putamen, amygdala, hippocampus, globus pallidus, and nucleus accumbens. Volume analyses of the subcortical regions were performed using FreeSurfer's automatic volumetric measurement methods (20). Thus, each subject' s dataset included structural data for a total of 82 brain regions, comprising both hemispheres.

### 2.5 Network construction

**Figure 1:**
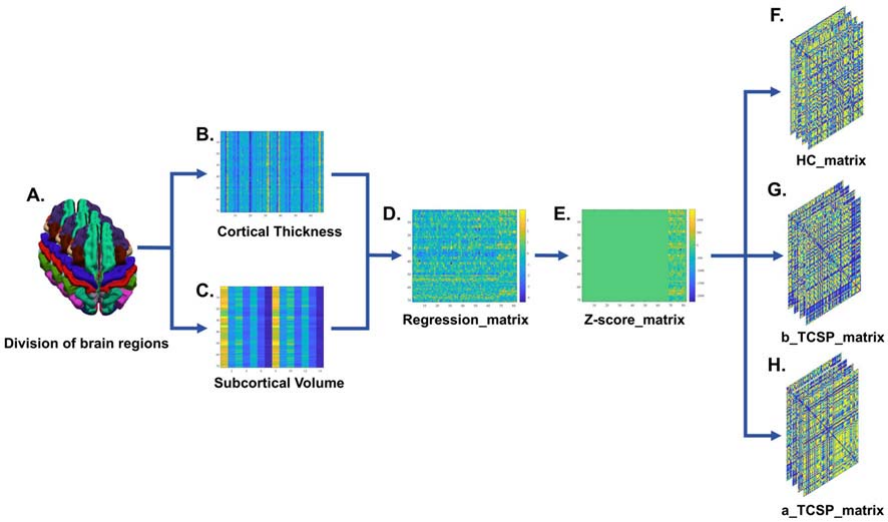
Schematic workflow for constructing individual-level brain structural covariance networks. **(A)** The brain is parcellated into 68 cortical regions and 14 subcortical regions based on the Desikan-Killiany atlas. **(B, C)** For each participant, cortical thickness is extracted for the 68 cortical regions and volumetric measures are obtained for the 14 subcortical regions. **(D)** Structural measures are adjusted for age, sex, and intracranial volume using regression analysis, generating corrected matrices for cortical and subcortical features. **(E)** Z-score normalization is applied to the corrected structural measures, resulting in standardized Z-score matrices. **(F-H)** Structural covariance values between regions are computed to construct structural covariance matrices. These matrices are generated separately for three groups: HCs, CNSP patients at baseline, and CNSP patients 3 months post minimally invasive intervention.

The network construction in this study was primarily based on the extracted cortical thickness from 68 ROIs and the volumes of 14 subcortical structures, with corrections for age, sex, and intracranial brain volume (21). First, the cortical thickness or subcortical volume for each individual's ROI (*X*_individual_) was calculated (Figures 1A–C). Next, the mean (μ) and standard deviation (σ) values of each ROI were determined for the HCs group. Subsequently, the residuals (*e*) for each individual's ROI were computed using the following formula (Figure 1D):


$$\begin{array}{l} \text{e}={\text{X}}_{\text{individual}}-\mu \end{array}$$

Further, z-scores were calculated to quantify the degree of brain morphological variation for each individual's ROI relative to the “HCs group mean.” A z-score greater than 0 indicates that the individual's measurement exceeds the average value of the HCs group, while a z-score less than 0 indicates that the individual's measurement is below the group's mean (Figure 1E):


$$\begin{array}{l} Z=\frac{e}{\sigma } \end{array}$$

Finally, the covariance (*x*) between the cortical and subcortical structures for each individual was calculated using the following formula (11, 22, 23) (Figures 1F–H):


$$\begin{array}{l} x=\frac{1}{\exp^{{\left({Z-score}_{thi}-{Z-score}_{thj}\right)}^{2}}} \end{array}$$

For each individual, a intra-individual structural covariance networks was constructed in whicheach ROI is treated as a node. The connection weights between nodes represent the similarity of Z-score deviations between ROI pairs within the sameindividual, rather than structural covariance across subjects. These network connections thus reflect the within-subject pattern of morphological deviation similarity among regions.

This similarity measure was chosen to capture coordinated morphological alterations specific to each subject; regions exhibiting similar deviations from the healthy population norm (e.g., concurrent atrophy or enlargement) are presumed to share pathological vulnerabilities or coupled developmental trajectories. Unlike traditional group-level covariance, these network connections thus reflect the within-subject pattern of morphological deviation similarity among regions, preserving individual heterogeneity that is critical for characterizing personalized disease patterns.

The intra-individual structural covariance networks were further thresholded based on network density and binarized. The thresholding criteria were determined according to the standards set by Uehara et al. (24) (1) more than 80% of nodes must be connected to other nodes; (2) modularity >0.3; (3) small-world > 1. This standard ensures consistency in the network features between individuals, enhancing the reliability of inter-group comparisons. The final network density range was determined as 0.1–0.37.

### 2.6 Network metrics

Within this network density range of 0.1-0.37, with a step size of 0.01, we calculated the individual covariance network metrics for each density level and computed the area under the curve (AUC) for each network metric. The estimation of global and nodal network topology properties was conducted using the Graph Theoretical Network Analysis (GRETNA) (25) package in MATLAB R2022b. These network metrics include:

(1) Global efficiency (measuring the efficiency of information transmission across all nodes in the network, reflecting the overall collaborative capacity of the network),
(2) Node clustering coefficient (indicating the density of actual connections between a node's neighbors, reflecting the tightness of the local network around that node),
(3) Shortest path length (the shortest connection path between any two nodes in the network, reflecting the minimum time or distance for information transmission),
(4) Node efficiency (measuring a node's ability to transmit information within the entire network, with higher values indicating greater contribution to the flow of information in the network),
(5) Node local efficiency (measuring the efficiency of information transmission within the neighborhood of a node, reflecting the effectiveness of the node's local network),
(6) Degree centrality (indicating the number of connections a node has, measuring the strength of its direct links to other nodes in the network),
(7) Betweenness centrality (measuring a node's ability to act as an intermediary in the network, reflecting its importance in connecting other nodes), and
(8) Small-world attributes, which include:
① Clustering coefficient (measuring the level of clustering among the neighbors of network nodes, reflecting the compactness of the network's local structure),
② Characteristic path length (the average shortest path length between all pairs of nodes in the network, reflecting the average efficiency of information propagation),
③ Normalized clustering coefficient Gamma (the ratio of the network's actual clustering coefficient to that of a random network, used to assess the network's clustering characteristics),
④ Normalized characteristic path length Lambda (the ratio of the network's actual characteristic path length to that of a random network, used to evaluate the global efficiency of information transmission),
⑤ Small-worldness Sigma (a measure of the balance between segregation and integration within the brain network) (26–28).

### 2.7 Statistical analysis

A non-parametric permutation test was conducted 10,000 times to assess the AUC of cortical morphology data (including 68 cortical thickness measures and 14 subcortical volume measures) and graph-theoretical network metrics between the patient group and HCs at baseline, as well as between the minimally invasive intervention group and HCs. Age and gender were included as covariates to account for their influence on the data. Multiple comparisons were corrected for using the false discovery rate (FDR) method. Subsequently, the AUCs of the network metrics showing significant differences were correlated with the percentage change in the Visual Analog Scale (VAS) scores before and after treatment in the patient group. Partial correlation analysis was performed, with age, gender, and pain duration considered as confounding variables, and adjustments for these variables were made in the analysis. The FDR method was used to correct for multiple comparisons, with a significance threshold set at *P* < 0.05.

## 3 Results

### 3.1 Demographics and clinical measures

A total of 33 CNSP patients were initially recruited for this study. However, 6 patients were lost to follow-up after the minimally invasive intervention (due to various reasons preventing their return to the hospital for subsequent assessments), and therefore, they could not undergo a second MRI or clinical data collection at the 3-month follow-up. Additionally, two patients were excluded during the data quality assessment due to excessive head motion. Consequently, the final sample comprised 25 CNSP patients, along with 25 HCs participants. There were no significant differences between the two groups in terms of age and gender (*P* > 0.05). The 25 CNSP patients included in the study showed a reduction in their VAS scores from 6.25 ± 1.12 to 2.26 ± 0.57 following the minimally invasive intervention. Table 1 provides detailed clinical and demographic data for both groups.

**Table 1:** Demographic and behavioral data.

**Characteristics**	**CNSP(*n* = 25)**	**HCs(*n* = 25)**	***P* value**
Gender (male/female)	12/13	12/13	1
Age (years)	48.38 ± 6.84	46.35 ± 8.76	0.235
Duration of pain (months)	37.35 ± 16.54	–	–
VAS (Baseline)	6.25 ± 1.12	–	–
VAS (after minimally invasive intervention therapy)	2.26 ± 0.57	–	–
Time interval between two scans (month)	3.47 ± 0.42	–	–

CNSP, chronic neck and shoulder pain; HCs, healthy controls; VAS, visual analog scale.

### 3.2 Longitudinal analysis of cortical thickness and subcortical volume

At baseline, the CNSP group exhibited significantly reduced cortical thickness compared to the HCs group in the following regions: left hemisphere (lh) bankssts (*P* = 0.045, FDR corrected), lh_inferior parietal (*P* = 0.041, FDR corrected), lh_isthmus cingulate (*P* = 0.007, FDR corrected), lh_medial orbitofrontal (*P* = 0.041, FDR corrected), lh_posterior cingulate (*P* = 0.001, FDR corrected), lh_superior temporal (*P* = 0.045, FDR corrected), lh_supramarginal (*P* = 0.042, FDR corrected), right hemisphere (rh) fusiform (*P* = 0.040, FDR corrected), rh_isthmus cingulate (*P* = 0.041, FDR corrected), rh_postcentral (*P* = 0.039, FDR corrected), rh_superior temporal (*P* = 0.038, FDR corrected), and rh_supramarginal (*P* = 0.046, FDR corrected).

**Figure 2:**
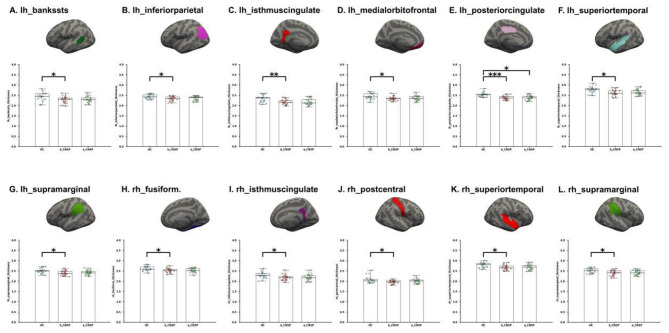
**(A-L)** Longitudinal changes in cortical thickness and subcortical volume in CNSP patients. CNSP patients exhibit a reduction in cortical thickness across 12 brain regions. Following 3 months of minimally invasive intervention, significant recovery is observed in all regions except for the posterior portion of the left cingulate gyrus. ^*^P<0.05; ^**^P<0.01; ^***^P<0.001.

Following minimally invasive intervention, the CNSP group showed partial normalization in cortical thickness across these regions. However, the lh_posterior cingulate (*P* = 0.0102, FDR corrected) continued to show a significant difference when compared to the HCs group (Figures 2A–L).

### 3.3 Network metrics analysis results

**Figure 3:**
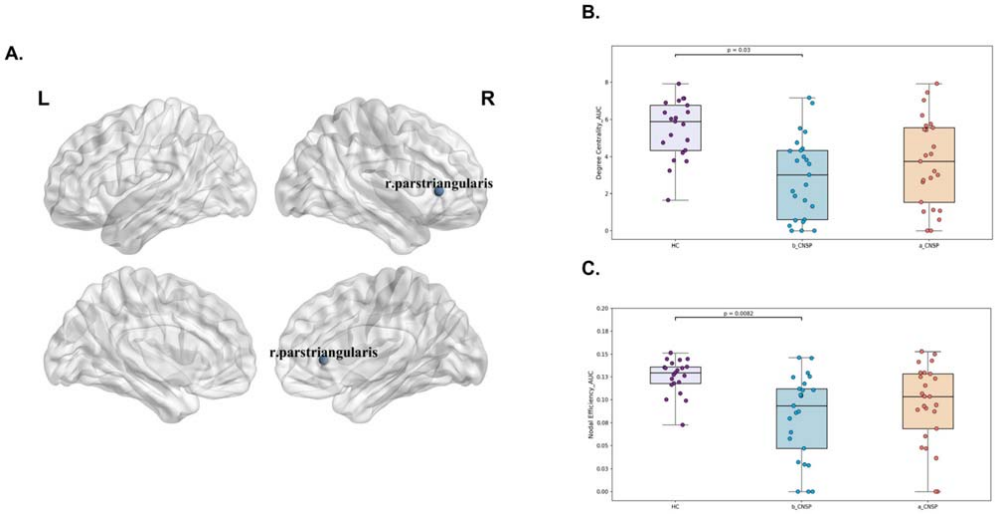
Analysis of local network features in the brain structural covariance network of CNSP patients. **(A)** Abnormal brain regions exhibiting altered local network features in CNSP patients. **(B)** Longitudinal changes in the Degree Centrality of the right pars triangularis in CNSP patients. **(C)** Longitudinal changes in the Nodal Efficiency of the right pars triangularis in CNSP patients.

Preoperative analysis of the CNSP group compared to the HCs group revealed significant reductions in local brain network characteristics as assessed by graph theory. Specifically, the rh_pars triangularis exhibited decreased Degree Centrality_AUC (*P* = 0.03, FDR corrected) and Nodal Efficiency_AUC (*P* = 0.0082, FDR corrected). Notably, these reductions showed significant longitudinal improvement at 3 months post-minimally invasive intervention (Figures 3A–C).

**Figure 4:**
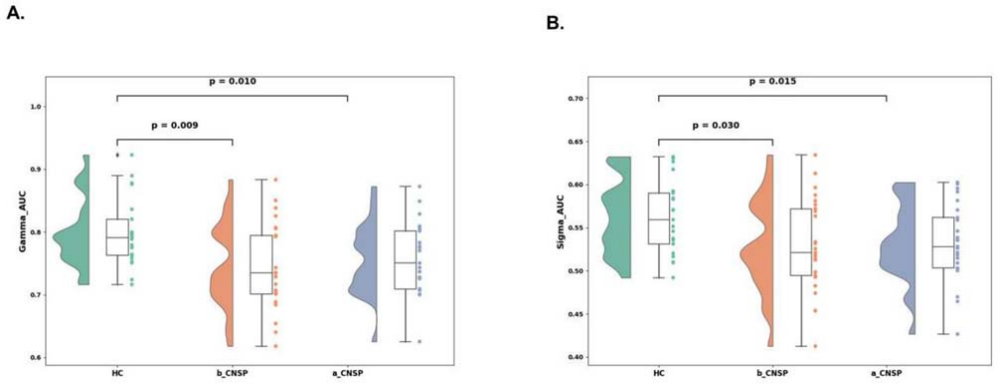
Analysis of global network features in the brain structural covariance network of CNSP patients. **(A)** Longitudinal changes in the small-world attribute Gamma in CNSP patients. **(B)** Longitudinal changes in the small-world attribute Sigma in CNSP patients.

In contrast to the changes observed in local metrics, global brain network characteristics, the CNSP group demonstrated significant reductions in small-world properties, specifically Gamma_AUC (*P* = 0.0093) and Sigma_AUC (*P* = 0.0301), compared to HCs. Unlike the local nodal metrics, no significant longitudinal changes were observed in these global metrics at 3 months following the intervention (Figures 4A, B).

### 3.4 Results of partial correlation analysis

**Figure 5:**
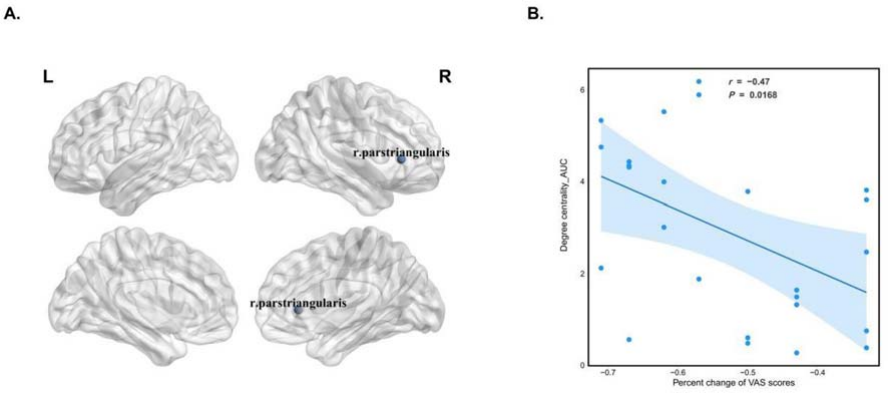
**(A, B)** Partial correlation analysis between baseline individual structural covariance network abnormalities and the percentage change in VAS scores before and after minimally invasive intervention in CNSP patients.

In an exploratory analysis, partial correlation analysis revealed that, at baseline, the Degree Centrality of rh pars triangularis in the CNSP group was negatively correlated with the percentage change in VAS scores (*r* = −0.47, *P* = 0.0168, FDR corrected) (Figures 5A, B).

## 4 Discussion

This study represents the first longitudinal application of individual structural covariance network analysis to patients with CNSP. Our results demonstrate that CNSP patients exhibit reduced cortical thickness and compromised nodal topology—specifically decreased Degree Centrality and Nodal Efficiency in the right inferior frontal gyrus (pars triangularis)—relative to healthy controls. Notably, minimally invasive intervention promoted partial recovery of these local morphological deficits, whereas global small-world properties (Gamma and Sigma) remained suppressed post-treatment. Furthermore, baseline centrality in the right inferior frontal gyrus was found to correlate with clinical pain relief, suggesting its potential prognostic value.

Regarding the longitudinal morphological changes, we observed that CNSP patients exhibited a decrease in cortical thickness (CT) across multiple brain regions associated with pain processing, a finding that partially aligns with the results reported by Woodworth et al. (29). While CT technically refers to the distance between the cortical surface and the underlying white matter, in the clinical context of chronic pain, a reduction in CT is not merely anatomical; it acts as a surrogate marker for neuroplastic maladaptation. Although MRI-based morphometry cannot directly resolve cellular mechanisms, we speculate that these reductions reflect underlying microscopic changes such as neuronal loss, synaptic pruning, or glial cell alterations driven by prolonged neural activity and continuous nociceptive input from the neck and shoulder region (30, 31). Crucially, our longitudinal data suggest that this atrophy is not permanent. Following minimally invasive intervention, cortical thickness in CNSP patients showed partial recovery. This structural restoration highlights the brain's inherent neuroplasticity—specifically, the potential for reorganization and repair once the chronic nociceptive drive is removed (32, 33). With the alleviation of pain symptoms, CNSP patients no longer need to process constant pain signals, reducing the allostatic load on the nervous system. Consequently, brain regions involved in pain processing may gradually return to their typical cortical morphology. Similar restorative trends have been observed in longitudinal studies of anxiety (34), severe depression (35), and chronic pancreatitis (36), suggesting that effective pain management can reverse maladaptive brain structural changes.

Turning to the specific topological alterations and their relevance to the pain experience, our study revealed that patients with CNSP exhibited decreased degree centrality (DC) and nodal efficiency (NE) in the right inferior frontal gyrus (rIFG, pars triangularis). Clinically, these graph theory metrics describe the “influence” and “speed” of a brain region within the whole network. Degree centrality reflects the number of direct connections (morphological coordination) a node has (37), while nodal efficiency assesses how rapidly a region exchanges information (38). The observed reduction in these metrics implies that in CNSP patients, the rIFG has become functionally “isolated” or less integrated with the rest of the brain. The inferior frontal gyrus is a critical hub for cognitive and affective functions, including emotional regulation, cognitive control, and decision-making (39). In the context of chronic pain, the rIFG is integral to the top-down modulation of pain signals. A loss of centrality and efficiency in this region suggests a disruption in the structural covariance between the rIFG and other pain-matrix areas (e.g., insula, cingulate cortex). This disruption likely impairs the patient's ability to cognitively inhibit pain or regulate the negative emotions associated with chronic neck and shoulder discomfort. Consequently, the pain processing may shift from a purely sensory phenomenon toward one that is more emotional and uncontrolled, thereby intensifying the subjective suffering. Although strictly inferring function from structure requires caution, these topological alterations likely underlie the inefficient processing of nociceptive signals associated with the persistence of pain symptoms.

Furthermore, we analyzed the global network properties to understand the overall brain efficiency. Gamma and Sigma are indices used to describe small-world networks, reflecting the balance between local clustering and global integration. Simply put, a high Gamma or Sigma indicates a “cost-effective” brain network that transmits information efficiently. In patients with CNSP, the observed reduction in Gamma and Sigma at baseline suggests a decline in this global efficiency (40, 41). This “loss of small-worldness” implies that the CNSP brain is less flexible and more energy-consuming when integrating nociceptive information. The reduced modularity (Sigma) indicates a disorganized network architecture, which may impair the coordination required to regulate continuous pain inputs effectively, leading to abnormal pain perception and maintenance of the chronic pain state.

Regarding the effects of the intervention and the specific role of the rIFG, our longitudinal results demonstrate that following minimally invasive interventions, the DC and NE in the rIFG (pars triangularis) recovered significantly. This finding identifies the rIFG as a key responsive target for pain relief. We interpret this recovery to mean that when chronic neck and shoulder pain is alleviated, the cognitive and emotional burden on the patient is reduced, allowing the rIFG to re-establish efficient connectivity (42). Specifically, the restoration of this region's topology suggests a recovery of the brain's “top-down” control mechanisms, enabling better emotional regulation and cognitive processing. However, interestingly, the global small-world properties (Gamma and Sigma) did not show significant post-intervention changes. We speculate this is because DC and NE reflect local nodal plasticity, which can recover rapidly as local circuits (like the rIFG) reorganize after pain relief (43). In contrast, Gamma and Sigma reflect global topological architecture (40). The neural network in CNSP patients may have undergone long-term, widespread remodeling45; thus, while local hubs like the rIFG recover quickly, the global network structure requires a longer duration to revert to a healthy “small-world” state.

Finally, the clinical translation of these findings is underscored by our correlation analysis. We found that at baseline, a higher Degree Centrality in the rIFG was negatively correlated with the percentage change in VAS scores (indicating better pain relief). This suggests that the rIFG connectivity profile may serve as a prognostic biomarker. Patients with higher baseline DC in the rIFG likely possess a more resilient neural network or greater “cognitive reserve,” enabling them to undergo more effective neuroadaptive changes during treatment (44). In other words, a better-integrated rIFG before surgery predicts a more robust recovery. This finding is clinically valuable, as measuring the connectivity of the rIFG could help clinicians assess a patient's potential for neuroplastic recovery and predict their response to minimally invasive interventions (45).

## 5 Limitations

In this study, although minimally invasive interventions for CNSP patients were consistently performed by a single physician using a standardized treatment protocol, and the analysis accounted for variables such as age, sex, and disease duration, treatment outcomes may still be influenced by other uncontrollable factors [e.g., the degree of cervical disc herniation, spinal nerve root inflammation, and postoperative care (46)]. Consequently, these findings should be further validated across different therapeutic approaches for CNSP and in diverse medical centers. Furthermore, we initially intended to construct a binary classification model based on the individual structural covariance network features of CNSP patients to predict their response to minimally invasive treatment. However, follow-up data revealed that, of the 25 CNSP patients included in the study, only 6 exhibited an insignificant treatment effect (with a VAS score reduction of less than 50%). This imbalance in the data, marked by a significant disparity between patients with substantial treatment effects and those with minimal effects, presents a challenge in developing and applying a reliable binary classification model using the current sample size (47). We therefore plan to enlarge the sample size in future studies and gather multicenter data to build a more robust predictive model and to conduct external validation of the model.

## 6 Conclusion

This study employed an individual structural covariance network approach to analyze structural MRI data of CNSP patients at baseline and 3 months post-minimally invasive intervention. The results revealed a reduction in Degree Centrality and Nodal Efficiency in the right inferior frontal gyrus triangular part of CNSP patients, which significantly recovered 3 months after the intervention. Additionally, global brain network analysis showed a decrease in small-world properties, specifically the Gamma and Sigma indices, but no significant recovery was observed 3 months following the minimally invasive intervention. Furthermore, correlation analysis indicated a significant relationship between the Degree Centrality of the right inferior frontal gyrus triangular part at baseline and the treatment efficacy of the minimally invasive intervention. These findings provide valuable longitudinal data for understanding the central mechanisms of pain in CNSP patients and identify potential biomarkers that may be used to predict the response to minimally invasive interventions in individuals with chronic cervical and shoulder pain.

## Data Availability

The datasets presented in this article are not publicly available due to privacy and ethical restrictions. Requests to access the datasets should be directed to the corresponding author Xiaoxue Xu, nclittlesnownc@163.com.
